# Novel Use of Inverted V-shaped High Tibial Osteotomy in Treating Valgus Knee via Double-Level Osteotomy: A Clinical Case Report

**DOI:** 10.7759/cureus.87146

**Published:** 2025-07-01

**Authors:** Shuzo Takazawa, Yuki Kato, Ken Ichikawa

**Affiliations:** 1 Sports Medicine, Kameda Medical Center, Kamogawa, JPN

**Keywords:** distal femoral osteotomy, double-level osteotomy, high tibial osteotomy, inverted v-shaped osteotomy, joint line obliquity, knee osteoarthritis, valgus knee

## Abstract

Valgus knee deformity presents a surgical challenge when severe, especially with a hip-knee-ankle (HKA) angle exceeding 10°. While double-level osteotomy (DLO) is often used to correct complex deformities, the application of inverted V-shaped high tibial osteotomy (iVHTO) in valgus knees is rare. We present the case of a 70-year-old man with lateral compartment osteoarthritis and HKA of −18°, treated with DLO combining medial closing wedge distal femoral osteotomy and iVHTO. A correction of 10° at the femur and 12° at the tibia was achieved, with final alignment aimed at a mechanical axis of 45%. At the one-year follow-up, radiographic parameters showed maintained correction and bone union, with improved Knee Injury and Osteoarthritis Outcome Score. This case suggests that iVHTO, although typically used in varus knees, can be safely and effectively applied in valgus deformity as part of a DLO strategy.

## Introduction

Valgus knee deformity is less common than varus alignment [1] and presents unique surgical challenges, particularly when severe, defined as a hip-knee-ankle (HKA) angle greater than 10° [2,3]. In these cases, single-level osteotomy may lead to excessive joint line obliquity (JLO), which can disrupt knee biomechanics, increase shear stress on cartilage surfaces, and compromise long-term joint function [4,5]. To overcome this, double-level osteotomy (DLO) involving corrections at both the distal femur and proximal tibia is used to achieve optimal mechanical alignment while minimizing JLO.

The HKA angle, a key indicator of coronal alignment, measures deviation of the mechanical axis. Additional parameters such as the mechanical lateral distal femoral angle (mLDFA) and medial proximal tibial angle (MPTA) help localize the origin of the deformity [6,7].

The choice of osteotomy technique depends on the anatomical location of the deformity [8]. Medial closing wedge distal femoral osteotomy (MCWDFO) is commonly used for femoral-based deformities [9,10], while medial closing wedge high tibial osteotomy (MCWHTO) is typically applied for tibial-based cases [9,11]. However, when both procedures are performed as closing wedge osteotomies, as a combination of MCWDFO and MCWHTO, the cumulative shortening of the limb can be significant, particularly in high-degree corrections.

Inverted V-shaped high tibial osteotomy (iVHTO) is a specialized technique originally developed for varus knees with severe medial compartment osteoarthritis. It offers several advantages over conventional methods, including bone preservation, intrinsic mechanical stability, and maintenance of the posterior tibial slope [12,13]. In iVHTO, two oblique cuts form a V-shaped configuration: a medial bone wedge is resected, and a controlled lateral opening achieves angular correction. Despite these benefits, its application in valgus knees remains virtually unreported.

To our knowledge, the use of iVHTO in combination with MCWDFO for severe valgus knee deformity has not been previously documented. This report presents a rare case successfully treated with this DLO approach and aims to demonstrate its feasibility and clinical utility for high-grade valgus alignment correction.

## Case presentation

**Figure 1:**
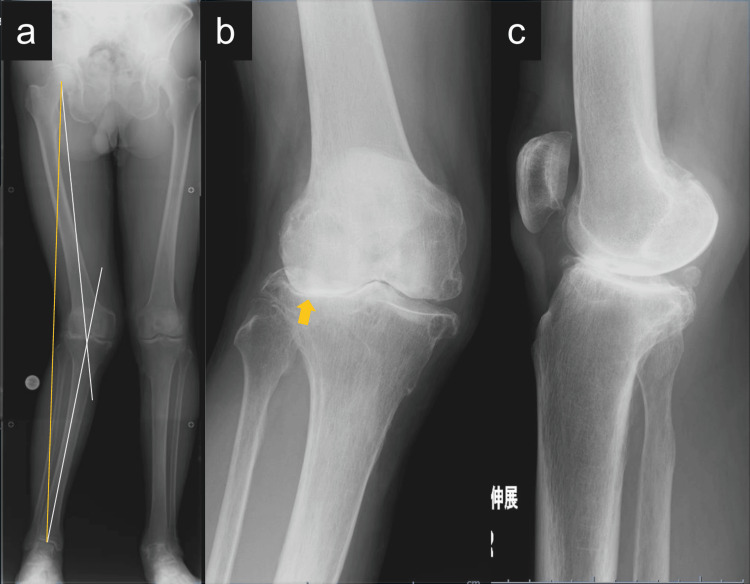
Preoperative radiographs of the right knee. (a) Standing full-length anteroposterior (AP) radiograph of the lower limbs showing severe valgus alignment of the right knee. The hip–knee–ankle angle was −18°, indicated by white lines. The mechanical axis, shown as a yellow line, deviates significantly laterally. (b) AP radiograph of the right knee highlighting joint space narrowing in the lateral compartment, marked by a yellow arrow. (c) Lateral radiograph showing joint line orientation and posterior tibial slope in extension.

A 70-year-old man presented with a 10-year history of progressive right lateral knee pain. He reported no history of trauma or previous surgery. On physical examination, valgus deformity and lateral joint line tenderness were observed. Preoperative radiographs revealed severe valgus alignment: HKA angle was −18°, the % mechanical axis (%MA) was 125%, mLDFA was 82°, MPTA was 94°, and joint line convergence angle (JLCA) was −8.7°. Radiographs also showed lateral joint space narrowing (Figure 1).

Preoperative Knee Injury and Osteoarthritis Outcome Score (KOOS) were as follows: symptoms, 57; pain, 61; activities of daily living (ADL), 69; sports/recreation, 25; and quality of life (QoL), 6.

The patient underwent DLO, consisting of MCWDFO and iVHTO, under general anesthesia without a tourniquet. Initial diagnostic arthroscopy confirmed lateral compartment osteoarthritis. A 10° correction was performed at the femur via MCWDFO, fixed with the TriS Medial DFO Plate System (Olympus Terumo Biomaterials Corp., Tokyo, Japan) and supported with a lateral TriS Small Plate (Olympus Terumo Biomaterials Corp.). Following the femoral osteotomy, a fibular osteotomy was performed through a posterolateral incision using an oblique saw cut, without fixation. The iVHTO was then performed via a straight anterolateral incision. The tibialis anterior and posteromedial structures were carefully released.

**Figure 2:**
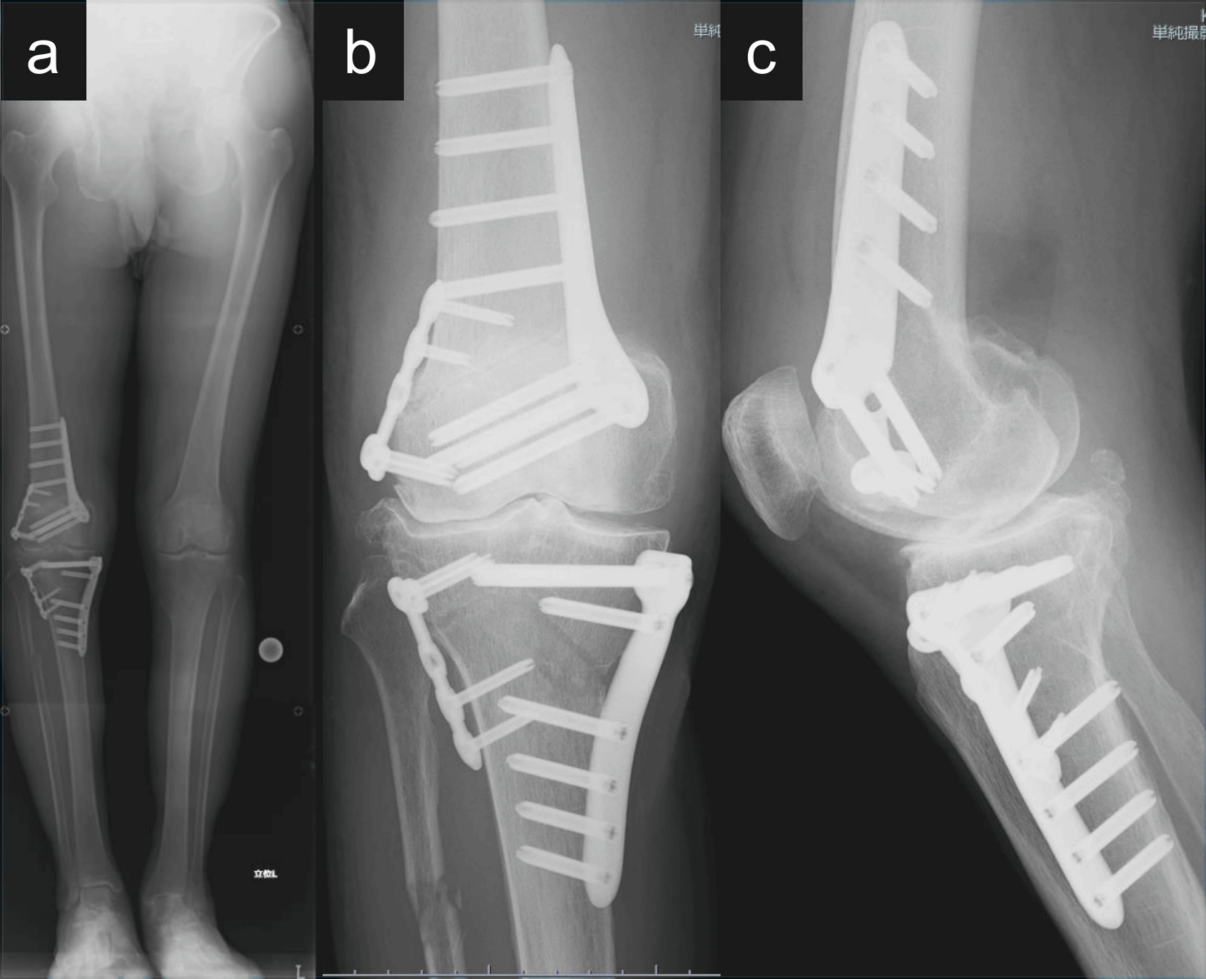
Immediate postoperative radiographs of the right knee following double-level osteotomy. (a) Standing full-length anteroposterior (AP) radiograph of the lower limbs demonstrating fixation after medial closing wedge distal femoral osteotomy and inverted V-shaped high tibial osteotomy. Internal fixation was achieved using the TriS Medial DFO Plate System (Olympus Terumo Biomaterial Corp., Tokyo, Japan) for the femoral side and the TriS Medial HTO Plate System (Olympus Terumo Biomaterial Corp.) for the tibial side. TriS Small Plates (Olympus Terumo Biomaterial Corp.) were additionally applied to the lateral aspects of both the femur and tibia to reinforce stability. (b) AP radiograph of the right knee displaying secure fixation of both osteotomy sites with clearly defined implant placement. (c) Lateral radiograph of the right knee showing well-aligned osteotomy lines and preservation of the posterior tibial slope.

Under fluoroscopy, the osteotomy apex was defined as a point 3 cm distal to the lateral intercondylar eminence. A protractor-installed wedge cutting guide (Olympus Terumo Biomaterials Corp.) was used to insert two pairs of Kirschner wires, guiding the osteotomy planes. A biplanar osteotomy was performed: a medial wedge was resected, and lateral cortical bone was cut. A 12° varus correction was achieved, and the resected bone was grafted into the lateral opening gap. Fixation was completed with the TriS Medial HTO Plate System (Olympus Terumo Biomaterials Corp.) and TriS Small Plate. Final alignment was confirmed using a mechanical axis rod, targeting a %MA of 45% (Figure 2).

At the one-year follow-up, the KOOS scores improved as follows: symptoms, 71; pain, 77; ADL, 80; sports, 60; and QoL, 43 (Table 1).

**Table 1:** Changes in KOOS before and after surgery. KOOS subscale scores improved across all domains at one year after double-level osteotomy. Notable improvements were observed in each subscale. KOOS: Knee injury and Osteoarthritis Outcome Score

KOOS Subscale	Preoperative score	Postoperative (1 year) score
Symptoms	57	71
Pain	61	77
Activities of Daily Living	69	80
Sports and Recreation	25	60
Quality of Life	6	43

The total KOOS score increased from 34.7 preoperatively to 77.2 at one year after surgery. All subscale improvements exceeded the minimal clinically important difference (MCID) of 8-10 points, indicating clinically meaningful gains in function, pain, and quality of life [14]. A comparison of radiographic parameters before and after surgery is shown in Table 2.

**Table 2:** Comparative radiographic parameters before and after surgery. Radiographic measurements show significant improvement in limb alignment one year postoperatively. The HKA angle shifted from −18° (severe valgus) to +6°. The %MA, initially located far lateral to the tibial plateau, was corrected to approximately 30% medially. Both the mLDFA and MPTA approached normal ranges, and JLCA was also reduced, reflecting balanced correction across femoral and tibial segments. HKA: hip–knee–ankle; %MA: mechanical axis; mLDFA: mechanical lateral distal femoral angle; MPTA: medial proximal tibial angle; JLCA: joint line convergence angle

Parameter	Preoperative score	Postoperative (1 year) score
HKA angle	−18°	+6°
% Mechanical Axis	125%	30%
mLDFA	82°	92°
MPTA	94°	84°
JLCA	−8.7°	−2.0°

**Figure 3:**
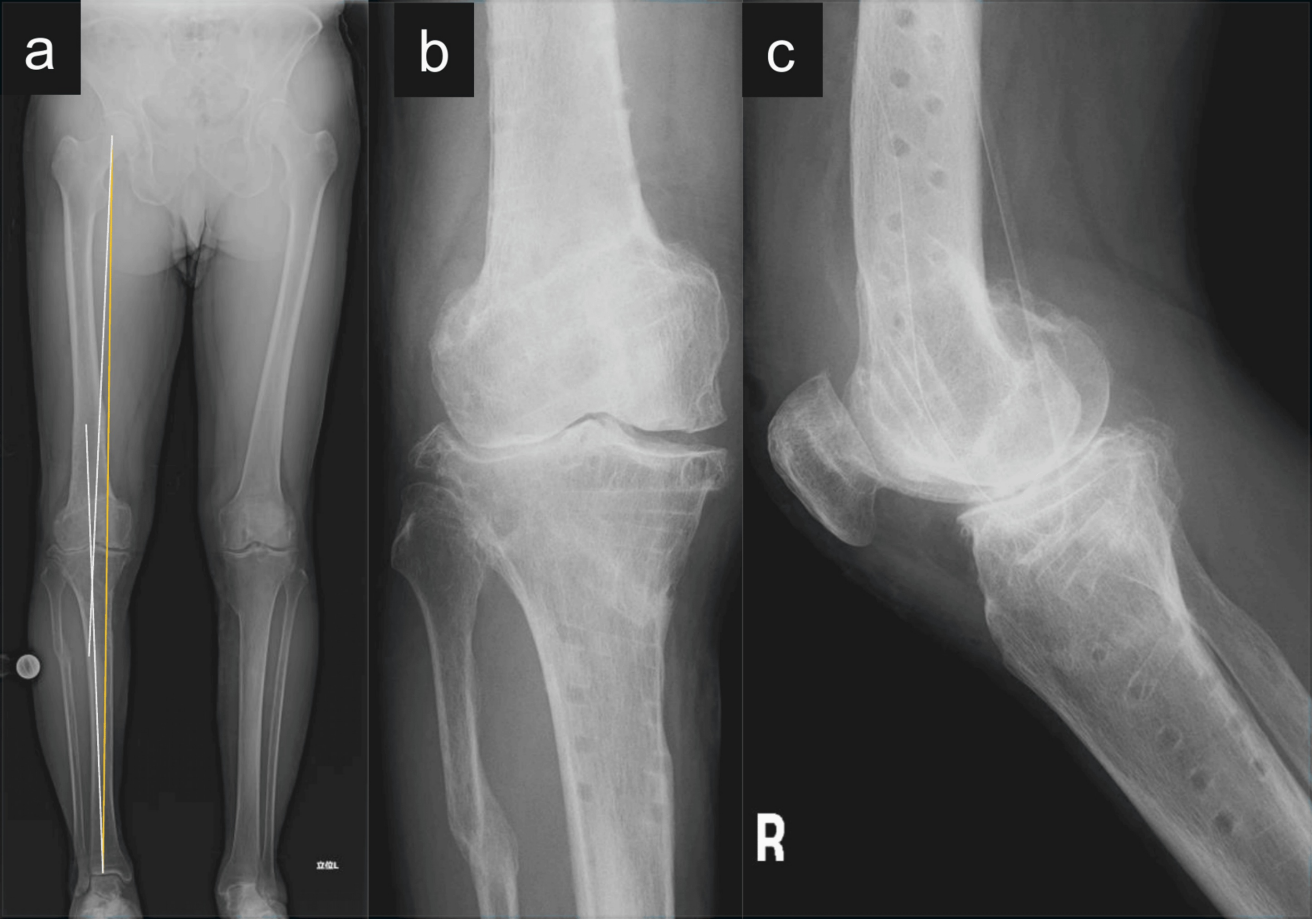
One-year postoperative radiographs of the right knee following double-level osteotomy. (a) Standing full-length anteroposterior (AP) radiograph showing maintained correction with an hip–knee–ankle angle of 6°, indicated by white lines. The mechanical axis, shown in yellow, passes through the medial 30% of the tibial plateau. (b) AP radiograph of the right knee demonstrating complete bone union at the femoral and tibial osteotomy sites. (c) Lateral radiograph confirming well-healed osteotomy lines and preservation of posterior tibial slope.

The preoperative HKA angle was −18°, indicating a severe valgus deformity. Postoperatively, the HKA improved to +6°, with a corresponding mechanical axis deviation corrected from approximately 125% to 30%. The mLDFA increased from 82° to 92°, and the MPTA was corrected from 94° to 84°, while JLCA improved from −8.7° to −2.0°. These changes reflect appropriate alignment correction distributed across both the femoral and tibial segments. Postoperative radiographs demonstrated good bone union and no correction loss (Figure 3).

**Figure 4:**
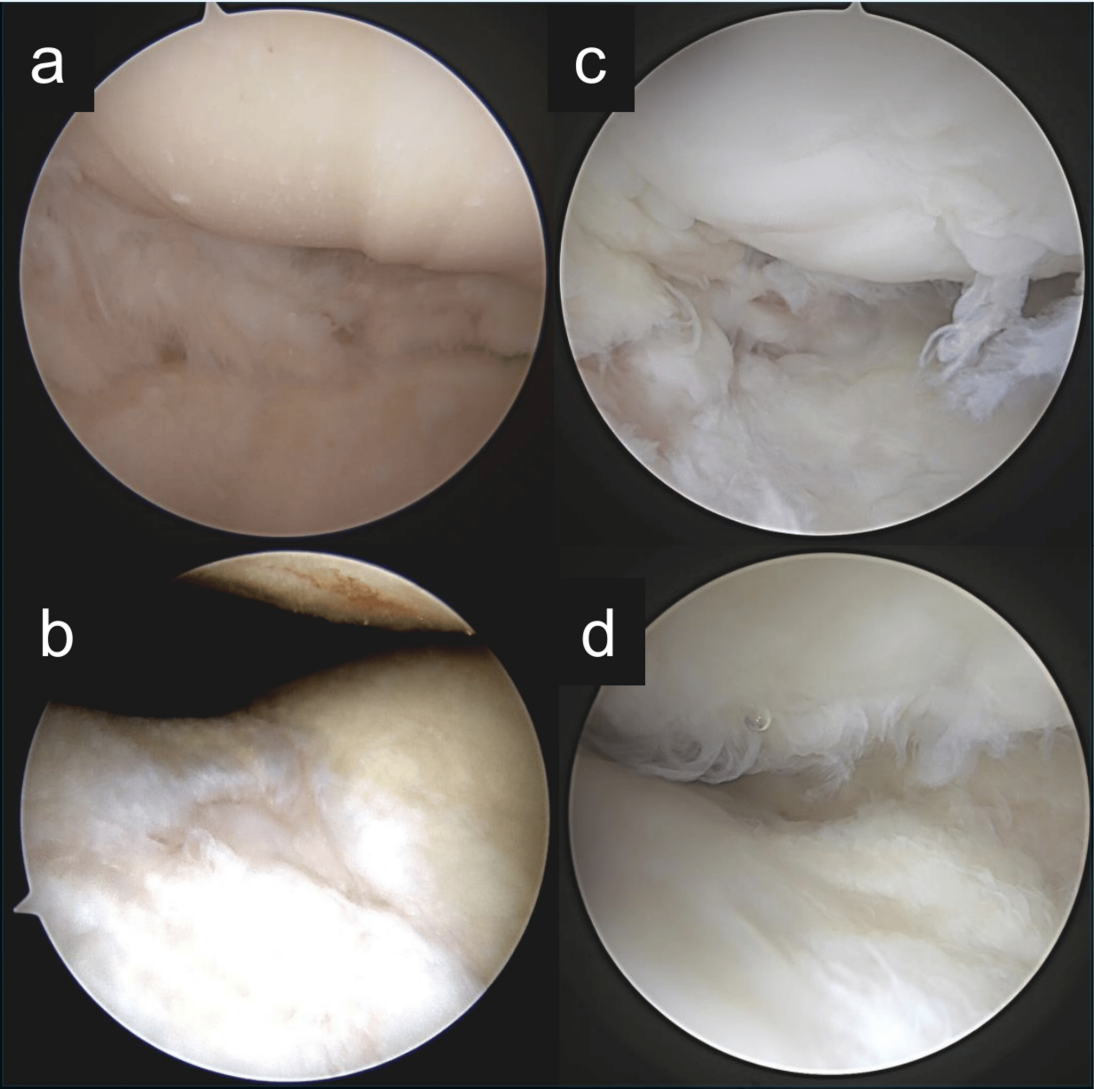
Arthroscopic findings at the time of initial surgery and at second-look arthroscopy after one year. (a, b) Arthroscopic views at the time of initial double-level osteotomy. (a) The lateral femoral condyle and lateral tibial plateau show full-thickness cartilage defects, classified as International Cartilage Repair Society (ICRS) Grade 4. (b) The trochlear groove demonstrates an ICRS Grade 4 cartilage defect on the femoral side. (c, d) Second-look arthroscopy performed at the time of implant removal, one year postoperatively. (c) The lateral compartment is covered with fibrocartilaginous tissue, indicating partial repair of the previous defect. (d) The trochlear cartilage surface is also covered with fibrocartilage, showing evidence of biological healing.

Partial weight-bearing was initiated at three weeks postoperatively, and full weight-bearing gait was allowed at five weeks. The implant was electively removed at one year postoperatively. During the procedure, second-look arthroscopy revealed that both the lateral femoral condyle and trochlear cartilage defects, previously graded as International Cartilage Repair Society (ICRS) Grade 4, were now covered with fibrocartilaginous tissue, indicating partial biological healing (Figure 4).

## Discussion

This case showed that DLO combining MCWDFO and iVHTO achieved effective alignment correction and functional improvement in a patient with severe valgus deformity. Fibrocartilaginous coverage observed at the one-year arthroscopy suggests early cartilage regeneration.

Severe valgus knee deformity presents distinct surgical challenges, especially when the HKA angle exceeds 10° [2]. Single-level osteotomy in such cases often results in excessive JLO, which may negatively affect joint mechanics and clinical outcomes [4,5,8]. In addition, when correction is performed through the distal femur alone, overstretching of the iliotibial band may occur, causing postoperative lateral thigh pain. DLO, which distributes correction between the femur and tibia [15-18], is therefore useful in minimizing both JLO and soft tissue strain.

For femoral-based valgus deformities, MCWDFO is commonly used, while MCWHTO is standard for the tibial side. However, combining two closing wedge osteotomies may result in considerable limb shortening. In this case, iVHTO was chosen on the tibial side to reduce bone resection and preserve limb length. Although iVHTO is more commonly used in varus knees [19,20], its application in valgus knees is extremely limited due to the technical difficulty of controlled lateral opening. In valgus alignment, the lateral tibial cortex is already under stress, increasing the risk of hinge instability or fracture. This requires meticulous surgical planning and precise technique.

Another key concern in high-grade valgus knees is soft tissue imbalance. Shortening of the lateral collateral ligament and laxity of the medial collateral ligament can make intraoperative alignment decisions particularly challenging [21]. Despite adequate bony correction, ligamentous imbalance can result in unintended overcorrection or residual malalignment. However, there are currently no standardized intraoperative metrics to guide soft tissue balance assessment, which remains a limitation in valgus knee osteotomy.

This case confirms the potential of MCWDFO combined with iVHTO as a viable DLO approach in severe valgus knees, particularly when considering limb length preservation and joint alignment. Further clinical reports and longer-term studies are needed to clarify its broader applicability and to develop reliable intraoperative indicators for soft tissue assessment.

## Conclusions

This case demonstrates that DLO combining MCWDFO and iVHTO can be an effective strategy for correcting severe valgus knee deformity. The use of iVHTO on the tibial side enabled controlled correction with minimal limb shortening and reliable fixation. Although iVHTO is rarely used in valgus knees, this case highlights its feasibility and potential value when incorporated into DLO. Further reports are needed to establish its role and optimal indications in the treatment of complex valgus deformities.
